# Peroxiredoxin Tsa1 Is the Key Peroxidase Suppressing Genome Instability and Protecting against Cell Death in *Saccharomyces cerevisiae*

**DOI:** 10.1371/journal.pgen.1000524

**Published:** 2009-06-19

**Authors:** Ismail Iraqui, Guy Kienda, Jérémie Soeur, Gérard Faye, Giuseppe Baldacci, Richard D. Kolodner, Meng-Er Huang

**Affiliations:** 1 UMR2027 Centre National de la Recherche Scientifique, Institut Curie, Université Paris Sud-XI, Orsay, France; 2 Ludwig Institute for Cancer Research, Department of Medicine and Cellular and Molecular Medicine, San Diego School of Medicine, University of California, La Jolla, California, United States of America; National Human Genome Research Institute, National Institutes of Health, United States of America

## Abstract

Peroxiredoxins (Prxs) constitute a family of thiol-specific peroxidases that utilize cysteine (Cys) as the primary site of oxidation during the reduction of peroxides. To gain more insight into the physiological role of the five Prxs in budding yeast *Saccharomyces cerevisiae*, we performed a comparative study and found that Tsa1 was distinguished from the other Prxs in that by itself it played a key role in maintaining genome stability and in sustaining aerobic viability of *rad51* mutants that are deficient in recombinational repair. Tsa2 and Dot5 played minor but distinct roles in suppressing the accumulation of mutations in cooperation with Tsa1. Tsa2 was capable of largely complementing the absence of Tsa1 when expressed under the control of the Tsa1 promoter. The presence of peroxidatic cysteine (Cys^47^) was essential for Tsa1 activity, while Tsa1^C170S^ lacking the resolving Cys was partially functional. In the absence of Tsa1 activity (*tsa1* or *tsa1^CCS^* lacking the peroxidatic and resolving Cys) and recombinational repair (*rad51*), dying cells displayed irregular cell size/shape, abnormal cell cycle progression, and significant increase of phosphatidylserine externalization, an early marker of apoptosis-like cell death. The *tsa1^CCS^ rad51*– or *tsa1 rad51*–induced cell death did not depend on the caspase Yca1 and Ste20 kinase, while the absence of the checkpoint protein Rad9 accelerated the cell death processes. These results indicate that the peroxiredoxin Tsa1, in cooperation with appropriate DNA repair and checkpoint mechanisms, acts to protect *S. cerevisiae* cells against toxic levels of DNA damage that occur during aerobic growth.

## Author Summary

Aerobically growing cells are continuously challenged by potent oxidants produced during normal cellular metabolism. These oxidants, including hydrogen peroxide and organic peroxides, are important components mediating various cell functions. However, they can also cause cell damage when present at toxic levels. Aerobic organisms possess extensive antioxidant systems to regulate oxidant levels. Among these, peroxiredoxins have received considerable attention in recent years as an expanding protein family involved in the enzymatic degradation of hydrogen peroxide and organic peroxides. To better understand the physiological role of the five peroxiredoxins in budding yeast *S. cerevisiae*, we performed a comparative study and found that one, Tsa1, played a key role in preventing DNA damage and assuring genome stability. Tsa1 also cooperated with other peroxiredoxins in antioxidant defense. These functions of Tsa1 required the presence of a cysteine at the catalytic site of this enzyme. Additional studies revealed that Tsa1 activity, in cooperation with appropriate DNA repair and checkpoint mechanisms, acts to protect cells against toxic levels of DNA damage that occur during aerobic growth.

## Introduction

Peroxiredoxins (Prxs), previously termed the thioredoxin peroxidases, have received considerable attention in recent years as a new and expanding family of thiol-specific antioxidant proteins [1],[2]. Prxs utilize cysteine (Cys) as the primary site of oxidation during the reduction of peroxides. Many organisms have multiple Prxs, with at least five Prxs having been identified in the yeast *Saccharomyces cerevisiae* and six in human cells [1],[3]. Prx isoforms are distributed differently within the cell. The reaction mechanisms of the Prxs have been established through extensive structural and biochemical analysis [2]. Based on structural and mechanistic data, Prxs can be divided into three subgroups as follows: 2-Cys Prx proteins, which contain both the N- and C-terminal-conserved Cys residues (peroxidatic and resolving Cys, respectively), and require both for catalytic function; atypical 2-Cys proteins, which contain only the N-terminal-conserved Cys but require one additional, nonconserved Cys residue for catalytic activity; and 1-Cys Prx proteins, which contain only the N-terminal Cys and require only this conserved single Cys for catalytic function. All three types of Prxs appear to share a similar catalytic mechanism, in which the peroxidatic Cys is oxidized to a sulfenic acid by either H_2_O_2_ or alkyl hydroperoxides [2]. In both types of 2-Cys Prxs, the sulfenic acid then reacts with the resolving Cys of the other subunit to form an intermolecular disulfide (typical 2-Cys Prxs), or with the C-terminal Cys of the same monomer to form an intramolecular disulfide (atypical 2-Cys Prxs). In both cases, the disulfides are specifically reduced by the thioredoxin and thioredoxin reductase system [2], with the exception of some prokaryotic Prxs, such as the *Escherichia coli* AhpC, which uses another protein, AhpF, for the regeneration of the reduced Prx [4]. In the 1-Cys Prxs, the sulfenic acid is directly reduced to a thiol, because there is no nearby Cys available to form a disulfide bond. The physiological source of the reducing equivalents for regenerating this thiol is not known. Upon exposure to high doses of H_2_O_2_, some Prxs suffer an oxidation of the peroxidatic Cys to sulfinic acid, which temporarily inactivates these enzymes. This hyperoxidation of 2-Cys Prx enzymes is reversible in cells by sulfiredoxin, an ATP-dependent reductases for Cys-sulfinic acid in Prxs [5],[6].

*S. cerevisiae* Tsa1 was the first characterized Prx, followed by Aph1 (cTpxIII) [7],[8]. Three additional isoenzymes, Tsa2, Prx1 (mTpx) and Dot5 (nTpx) were then identified and characterized [3]. Tsa1 and Tsa2 are typical 2-Cys Prxs, Ahp1 and Dot5 are atypical 2-Cys Prxs and Prx1 is a 1-Cys Prx. More recently, three glutathione peroxidase homologues Gpx1, Gpx2 and Gpx3 that are similar to mammalian phospholipid hydroperoxide glutathione peroxidases and thought to use glutathione as an electron donor [9],[10], were shown to encodes atypical 2-Cys Prxs that preferably use thioredoxin as an electron donor [11],[12]. However, these Gpx-like enzymes are phylogenetically distinct from Tsa1, Tsa2, Ahp1, Prx1, Dot5 [11] and therefore have not been studied here.

The five *S. cerevisiae* Prxs have different sub-cellular localizations: Tsa1, Tsa2 and Ahp1 are localized in the cytoplasm; Prx1 is localized in mitochondria; and Dot5 is localized in the nucleus [3]. Tsa1 and Tsa2 are highly homologous. It is unknown whether the sub-cellular localization of the Prxs changes in response to oxidative stress. All Prxs exert thioredoxin-dependent peroxidase activity while the specific peroxidase activities of five Prxs toward various peroxides are different. Tsa1, Tsa2 and Prx1 preferentially reduce H_2_O_2_ rather than alkyl hydroperoxide, whereas Ahp1 and Dot5 showed the reverse selectivity [3]. Tsa2 also has peroxynitrite reductase activity [13], pointing its role in the protection against nitrosative stress. In addition to their peroxidase activity, both Tsa1 and Tsa2 have been shown to act as a molecular chaperone that promotes resistance to heat shock [14]. Their chaperone activity involves a stress-dependant switch from lower molecular weight forms to higher molecular weight complexes.

The transcriptional levels of the *PRX* genes are also quite different from each other. There are discrepancies in the reported relative order of mRNA abundance; however, most studies show that *TSA1* mRNA is the most abundant *PRX* mRNA at different growth stages, followed by *AHP1* [3],[15]. Consistent with this, the order of absolute protein levels, estimated by large-scale tandem affinity purification analysis is Tsa1>Ahp1>Tsa2>Prx1>Dot5. Tsa1 is presents in approximately 3.8×10^5^ molecules per log phase cell, higher than Ahp1 which is present at approximately 1.6×10^4^ molecules per log phase cell and much higher than Tsa2, Prx1 and Dot5 which are present at about 1.8–4.8×10^3^ molecules per log phase cell [16]. The transcription of the *PRX* genes are usually upregulated to various degrees in response to treatment with various oxidants and *TSA2* is the most strikingly upregulated of the *PRX* genes [3],[15],[17]. However, Tsa1 is always the most abundant of the Prx proteins due to the significantly higher basal transcription level of the *TSA1* gene [15].

The physiological role of each Prx has been explored. Notably, Tsa1 has been shown to be a significant contributor to genome stability; its absence leads to a broad spectrum of spontaneous mutations and genome rearrangements that are thought to result from increased oxidative damage to the DNA [18]. Other *PRX* genes only play a minor role in the suppression of genome instability, but their deletion increases the spontaneous mutation frequency of a *tsa1* mutant [19]. Importantly, mutations affecting recombinational repair including *rad51* or *rad52*, or the Rad6-mediated post-replication repair pathway including *rad6*, cause *tsa1* cells to be very sick or to die rapidly under aerobic conditions but not anaerobic conditions, emphasizing the importance of Tsa1 for genome protection [20]. The ability of other Prxs to promote the viability of aerobically grown cells deficient in recombinational repair is unknown. Consistent with this, *tsa1* mutants exhibit reduced clearance of reactive oxygen species (ROS) induced by exogenous treatment with H_2_O_2_ [13]. In contrast to the predominant role of Tsa1, Tsa2 seems to cooperate with Tsa1 in the cellular defense against reactive oxygen and nitrogen species [13]. Ahp1, the predominant cytoplasmic alkyl-hydroperoxide reductase, and Dot5, the nuclear isoform, appear to be necessary for maintaining cellular viability at stationary phase cells in which glucose as a carbon source is depleted and *S. cerevisiae* cells begin to use fatty acids as energy sources [3],[21]. In this situation, organic hydroperoxides, such as fatty acid hydroperoxide, should accumulate to high levels in cells. Prx1 has a specific protective role towards peroxides produced during respiration, consistent with its mitochondrial localization [3],[22]. *S. cerevisiae* Prxs are only known to have stress-protective functions in contrast to the unique *Schizosaccharomyces pombe* Prx, Tpx1, which is also involved in H_2_O_2_ signaling [23],[24]. Surprisingly, mutants in which *TSA1*, *TSA2*, *AHP1*, *PRX1* and *DOT5* are all deleted are still viable [19], which contrasts with the lethality of the *S. pombe* mutant lacking Tpx1 [25]. Taken together, it is reasonable to assume that each *S. cerevisiae* Prx plays a specific role, defined by its cellular location, substrate specificity and expression pattern. On the other hand, different Prxs might cooperate with each other in antioxidant defense.

To gain more insight into the physiological role of the *S. cerevisiae* Prxs, we performed a comparative study of Prxs for their respective role in maintaining the genome stability and in sustaining aerobic viability of cells deficient in recombinational repair. Tsa1 played a key role in this regard while Tsa1 and Dot5 cooperated to suppress DNA damage that leads to gross chromosomal rearrangements (GCRs). Tsa2 shared functional similarity with Tsa1 and was capable of largely complementing the absence of Tsa1 when expressed under the control of Tsa1 promoter. Peroxidase active site was essential for Tsa1 to function in suppressing genome instability and cell death. Finally we addressed the fate of mutant cells with compromised Tsa1 activity and deficient recombinational repair.

## Results

### Tsa1 Is the Major Prx and Cooperates with Other Prxs in Antioxidant Defense

As previously observed [18], deletion of *TSA1* resulted in elevated mutation rates as determined by the Can^r^ assay that detects all gene inactivating mutations that can occur in the 1.8-kb *CAN1* gene, which are most frequently base substitution and frameshift mutations, the *hom3-10* assay that detects reversion of a +1 insertion in the *HOM3* gene, the *lys2-Bgl* assay that detects reversion of a +4 insertion in the *LYS2* gene, and the GCR assay that measures gross chromosomal rearrangements, indicating the importance of Tsa1 in preventing a broad spectrum of types of genomic instability. Deletion of *TSA2*, *AHP1*, *PRX1* or *DOT5* did not cause a mutator phenotype that could be detected with these mutator assays, confirming the unique role of Tsa1 in maintaining genome stability (Table 1). To address whether Tsa1 might share some overlapping function with other Prxs in this regard, we constructed double mutants combining *tsa1* and a mutation of one of other four Prxs. The *tsa1 tsa2* double mutant showed an increase in the Can^r^, *hom3-10* and *lys2-Bgl* mutation rates relative to the *tsa1* and *tsa2* single mutants (95% confidence limits), whereas the GCR rate of the double mutant appeared to be similar compared to the *tsa1* mutant. However, the Can^r^, *hom3-10* and *lys2-Bgl* rates of the *tsa1 tsa2* double mutant were not much higher than the sum of the rates of each single mutant (95% confidence limits). Simultaneous elimination of *TSA1* and *AHP1*, or *TSA1* and *PRX1* did not appear to change the mutation rates in the four assays compared to *tsa1* single mutant. The Can^r^, *hom3-10* and *lys2-Bgl* mutation rates of the *tsa1 dot5* double mutant appeared to be only slightly higher compared to *tsa1* single mutant. In contrast, the GCR rate of the *tsa1 dot5* double mutant showed a significant increase relative to the *tsa1* and *dot5* single mutants (Table 1). Taken together, these analyses confirmed the predominant role of Tsa1 in suppressing mutations; however, Tsa2 likely shared some backup overlapping function with Tsa1 in preventing base substitution and frameshift mutations while Tsa1 and Dot5 cooperated to prevent GCRs.

**Table 1:** Mutation rates of peroxiredoxin mutants.

Strain	Relevant genotype	Can^r^ rate (×10^−7^)	Hom^+^ rate (×10^−8^)	Lys^+^ rate (×10^−8^)	GCR rate (×10^−10^)
RDKY3615	Wild-type	4.2 (3.5–4.9)	0.9 (0.5–1.4)	1.2 (0.9–1.4)	4.0 (2.6–7.0)
RDKY5502	*tsa1*	40.6 (34.9–45.6)	5.4 (4.1–5.7)	10.9 (10.3–14.3)	60.1 (37.1–96.2)
RDKY5653	*tsa2*	6.7 (5.4–7.5)	0.9 (0.6–1.5)	1.8 (1.8–3.1)	ND
RDKY5654	*ahp1*	6.6 (5.5–7.5)	1.3 (0.9–1.6)	2.4 (1.8–3.9)	ND
RDKY5655	*prx1*	5.6 (4.8–6.7)	1.2 (0.6–2.0)	1.8 (1.2–2.6)	ND
RDKY5656	*dot5*	5.8 (5.1–7.0)	1.7 (1.1–2.9)	1.4 (1.2–1.7)	7.1 (4.0–11.6)
RDKY5696	*tsa1 tsa2*	58.0 (53.0–61.7)	7.7 (6.4–9.0)	17.7 (16.1–19.9)	73.9 (49.0–148.0)
RDKY5697	*tsa1 ahp1*	33.8 (31.7–39.2)	4.1 (3.5–6.4)	8.3 (7.6–9.8)	49.9 (35.3–91.3)
RDKY5698	*tsa1 prx1*	38.3(34.2–40.8)	7.6 (6.2–10.4)	15.2 (13.4–16.2)	67.0 (50.5–154.2)
MEHY793	*tsa1 dot5*	54.1 (45.4–57.2)	8.7 (5.3–9.6)	15.7 (11.4–20.9)	344.9 (216.4–780.8)
MEHY1710	*tsa1*::*TSA2*	14.5 (12.6–17.7)	3.2 (1.7–4.2)	4.2 (2.6–5.3)	6.3 (2.5–11.8)
MEHY1702	*tsa1^C47S^*	40.7 (36.3–42.2)	7.6 (5.5–8.6)	12.5 (9.9–13.1)	52.3 (26.5–126.1)
MEHY1593	*tsa1^C170S^*	11.7 (9.8–12.4)	2.2 (1.6–3.1)	4.4 (3.6–5.1)	8.3 (3.8–16.9)
MEHY1590	*tsa1^CCS^*	41.1 (37.8–48.8)	6.7 (5.2–9.2)	12.5 (11.2–13.4)	47.5 (27.7–69.9)

The numbers in parentheses indicate the low and high values for the 95% confidence interval for each rate obtained.

ND, not precisely determined. We did not analyze enough events to determine the GCR rates of these mutants but estimated that they were similar to that of the wild-type strain.

**Figure 1:**
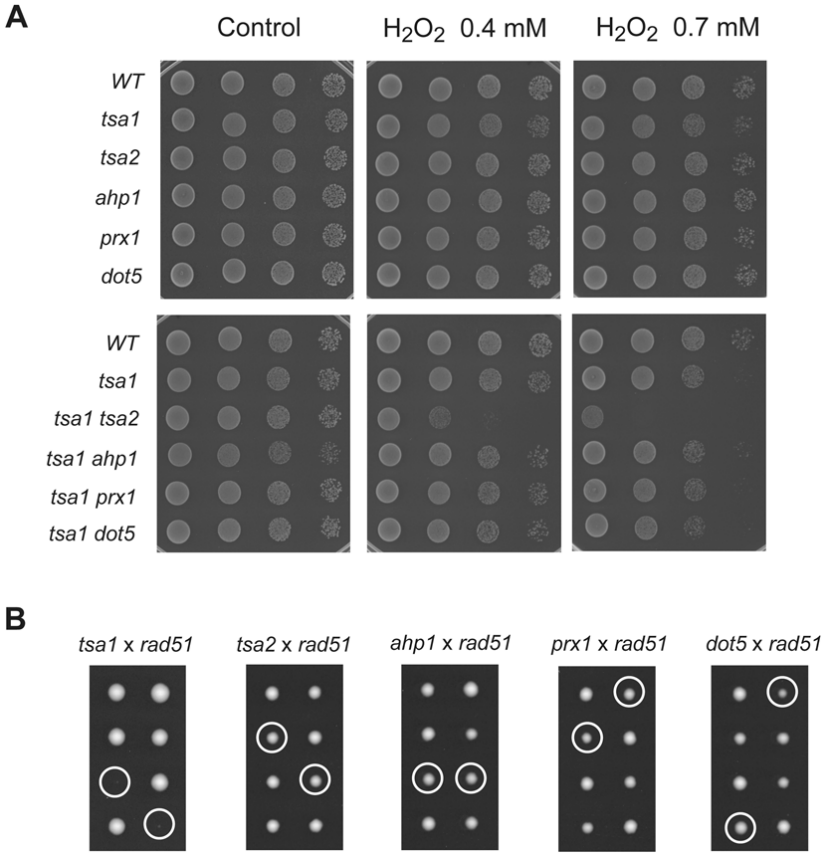
Comparative study of the five Prxs. (A) Sensitivity of *S. cerevisiae prxs* mutant strains to H_2_O_2_. Equal numbers of cells were serially diluted (10 fold per dilution) and spotted onto SC medium or SC medium containing indicated concentration of H_2_O_2_. (B) Effect of inactivation of *RAD51* upon viability and growth of each *prx* mutant. A *rad51* strain (MEHY694) was crossed with *tsa1*, *tsa2*, *ahp1*, *prx1*, and *dot5* single mutants to form heterozygous diploids. After sporulation of these heterozygous diploids, tetrads were dissected and grown on YPD at 30°C for 3 days. Spore genotypes were determined by replica plating on appropriate media. Representative tetrads are presented. Circles indicate either the inferred or determined *prx* and *rad51* double mutants.

To determine whether Tsa1 might share some overlapping function with other Prxs in the cellular defense against exogenous oxidative stress, we assessed sensitivity of above mentioned *prx* single and double mutants to H_2_O_2_ in a spot assay (Figure 1A). The *tsa1* mutant was weakly sensitive to H_2_O_2_ whereas the *tsa2*, *ahp1*, *prx1* and *dot5* single mutants had the same sensitivity as wild-type cells. The *tsa1 tsa2* double mutant was much more sensitive to H_2_O_2_ than either single mutant and the *tsa1 ahp1*, *tsa1 prx1 and tsa1 dot5* double mutants showed similar sensitivity to the *tsa1* single mutant. This suggests that Tsa2 cooperate with Tsa1 in antioxidant defense against exogenous oxidative stress.

We have previously shown that combining a *tsa1* mutation with *rad6* or *rad51* mutations, among others, results in cell death [20]. The synthetic lethality of these double mutation combinations is likely due to excessive ROS-related DNA lesions that occur in the absence of *TSA1* in combination with reduced repair, as anaerobic growth conditions restore cellular viability of these double mutants [26]. In order to assess whether the absence of other four Prxs could produced ROS-related DNA lesions that require recombinational DNA repair for cellular survival, we analyzed the meiosis products of heterozygous diploid strains containing *rad51* allele and one *prx* deletion allele. Tetrad dissection and consequent genotype analysis confirmed that *tsa1 rad51* double mutations were lethal and could only form microcolonies while none of other *prx* deletion alleles in combination with a *rad51* mutation resulted in lethality (Figure 1B). However, a slight slow growth was usually visible for all four viable double mutants at early stages of growth comparing with neighbor wild-type or single mutant colonies and this difference became less evident after 3-days of incubation of the tetrad dissection plates at 30°C. Thus, we concluded that only *tsa1* cells produce high enough levels of DNA damage to require recombinational DNA repair for cellular survival. This phenotype distinguishes Tsa1 from the other *S. cerevisiae* Prxs.

### Expression of *TSA2* under the Control of *TSA1* Promoter Restores the Genome Stability of a *tsa1* Strain

**Figure 2:**
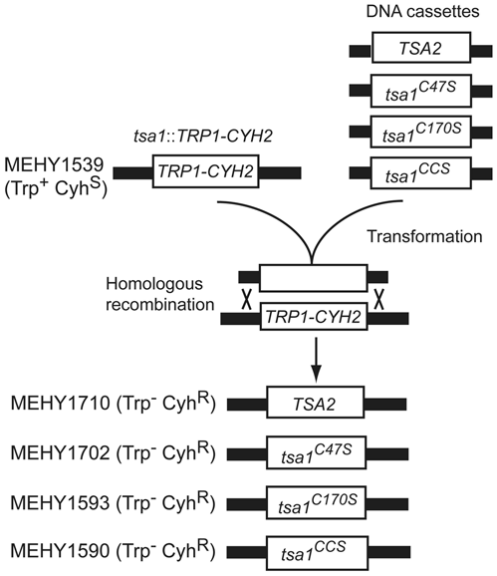
Construction of the strains expressing desired genes under control of the *TSA1* promoter. The *S. cerevisiae* strains expressing *TSA2* or mutated forms of *TSA1* (*tsa1^C47S^*, *tsa1^C170S^*, and *tsa1^CCS^*) under control of the endogenous *TSA1* promoter were constructed as described in Materials and Methods. The desired coding sequence was tailed by PCR with sequence homologous to regions flanking the chromosomal *TSA1* to allow targeted recombination.

**Figure 3:**
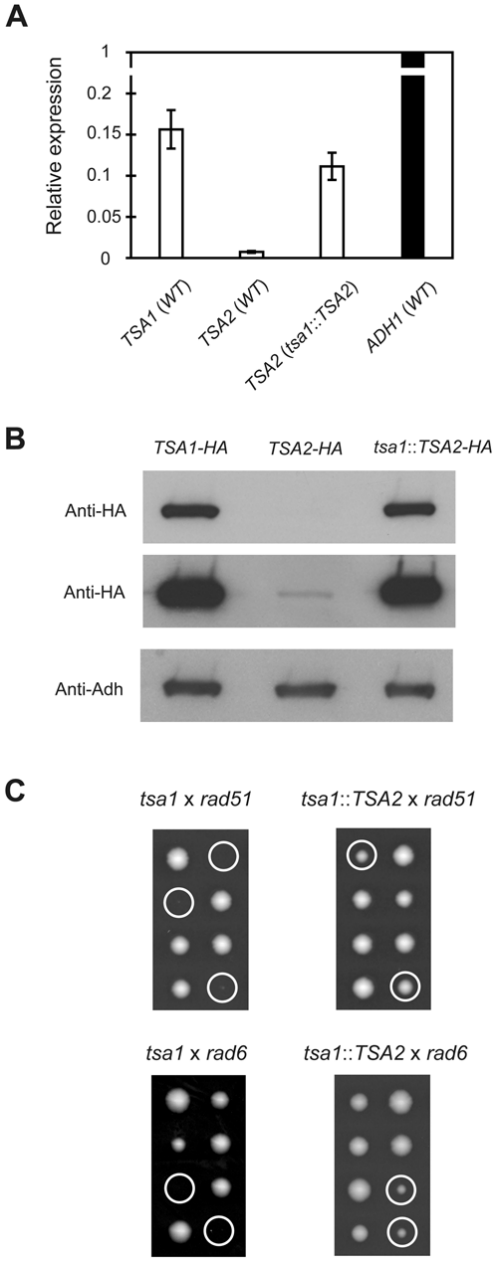
Effect of *TSA2* expression under the control of the *TSA1* promoter. (A) Analysis of mRNA expression by quantitative RT-PCR. For each experiment, the relative levels of *TSA1* or *TSA2* relative to *ADH* were calculated with the *ADH* level set as 1. *Columns*, mean of 3 independent experiments; *bars*, SD. (B) Analysis of protein level by Western blot. 3HA-tagged native Tsa1, native Tsa2 and *tsa1*::*TSA2* produced Tsa2 were detected by anti-HA antibody staining with short (upper panel) and long (middle panel) exposures presented and Adh levels (encoded by *ADH1*) as a loading control (bottom panel). (C) The *tsa1* (RDKY5502) and *tsa1*::*TSA2* (MEHY1710) mutants were crossed with *rad51* (MEHY694) and *rad6* (MEHY575) mutants and the spore clones obtained by tetrad dissections of the resulting heterozygous diploids (MEHY101 *tsa1*/*TSA1 rad51*/*RAD51*, MEHY1838 *tsa1*::*TSA2*/*TSA1 rad51/RAD51*, MEHY591 *tsa1/TSA1 rad6/RAD6* and MEHY1841 *tsa1*::*TSA2/TSA1 rad6/RAD6*) are shown. Circles indicate either the inferred or determined *tsa1 rad51* or *tsa1*::*TSA2 rad51* double mutants (upper panel) and *tsa1 rad6* or *tsa1*::*TSA2 rad6* double mutants (bottom panel).

Given the importance of Tsa1 in maintaining genome stability and cell survival, and that Tsa1 and Tsa2 are both cytoplasmic and share a high degree of sequence identity, we explored the ability of Tsa2, when expressed under the control of the Tsa1 promoter to complement defects caused by a *tsa1* mutation. Consequently, the *tsa1*::*TSA2* strain in which the *TSA1* gene (ATG-Stop) was replaced by the *TSA2* coding sequence was constructed as illustrated in Figure 2. Quantitative real-time reverse transcription PCR (RT-PCR) showed that the level of *TSA2* mRNA was 5% of that of *TSA1* mRNA in log phase wild-type cells but increased to 70% of the wild-type *TSA1* mRNA levels in the *tsa1*::*TSA2* strain (Figure 3A). To compare the protein levels of native Tsa1, native Tsa2 and *tsa1*::*TSA2* strain-produced Tsa2, we tagged the C-terminal ends of each protein with an influenza virus hemagglutinin (HA) epitope tag, enabling the immunodetection of these proteins with the same anti-HA antibody. The protein level of Tsa1 was much higher than that of Tsa2 in wild-type cells while Tsa2 produced in the *tsa1*::*TSA2* strain was comparable to the physiologic level of Tsa1 (Figure 3B). Taken together, we concluded that the *tsa1*::*TSA2* strain-produced *TSA2* mRNA and protein are comparable to the physiological level of *TSA1* transcript and protein.

We then examined the ability of the expression of Tsa2 to complement the phenotypes of a *tsa1* strain. The mutation rates of the *tsa1*::*TSA2* strain in all four mutator assays were significantly reduced compared the mutation rates of the *tsa1* mutant (95% confidence limits), but were higher than the mutation rates of the wild-type strain (Table 1). We next tested the ability of Tsa2 expressed under the control of the *TSA1* promoter to complement the synthetic lethality of *tsa1 rad51* and *tsa1 rad6* mutants. Four diploid strains (*tsa1*/*TSA1 rad51*/*RAD51*, *tsa1*::*TSA2*/*TSA1 rad51*/*RAD51*, *tsa1*/*TSA1 rad6*/*RAD6*, and *tsa1*::*TSA2*/*TSA1 rad6*/*RAD6*) were sporulated and dissected, and the spore dissection plates were incubated under aerobic conditions for four days. As previously shown, a *tsa1* mutation in combination with a *rad51* mutation resulted in lethality (Figure 1B, Figure 3C). In contrast, spore products from diploid *tsa1*::*TSA2/TSA1* strains showed an increased frequency of viable spores, indicating complementation. Genotyping of spore colonies confirmed that *tsa1*::*TSA2 rad51* mutants were viable, although these mutant colonies were smaller than those of the *rad51* single mutant (Figure 3C). A *tsa1* mutation in combination with a *rad6* mutation also resulted in lethality as previously reported [20], and viability was largely restored by expression of Tsa2 from the *tsa1*::*TSA2* strain (Figure 3C). Therefore, expression of Tsa2 under the control of *TSA1* promoter complemented the synthetic lethality of *tsa1 rad51* and *tsa1 rad6* double mutants. Taken together, these results indicate that Tsa2 produced at higher levels under the control of the *TSA1* promoter was capable of largely replacing the function of Tsa1 in regard to suppression of genome instability and synthetic lethality.

### Peroxidase Active Site Is Essential for Tsa1 to Function in Suppressing Genome Instability and Cell Death

**Figure 4:**
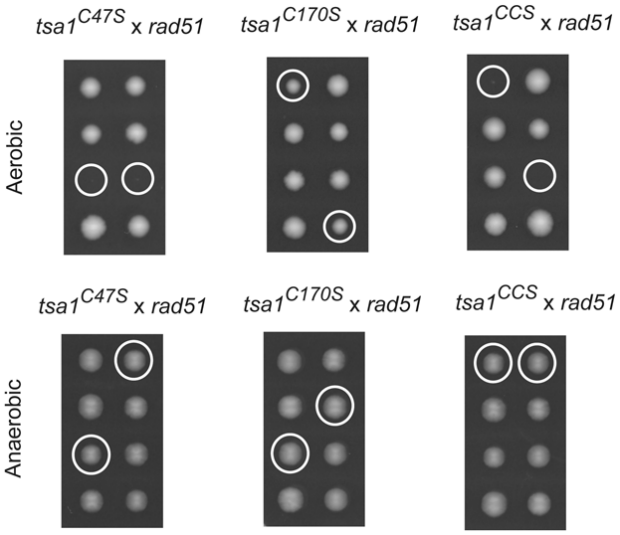
Effect of Cys substitutions on Tsa1 function in suppressing cell death. The *tsa1^C47S^*, *tsa1^C170S^*, and *tsa1^CCS^* mutants were crossed with a *rad51* single mutant, tetrads of resulting heterozygous diploids (MEHY1844 *tsa1^C47S^*/*TSA1 rad51/RAD51*, MEHY1847 *tsa1^C170S^/TSA1 rad51/RAD51*, and MEHY1850 *tsa1^CCS^*/*TSA1 rad51/RAD51*) were dissected and then incubated under aerobic or anaerobic conditions for 3 or 5 days respectively. Circles indicate either the inferred or determined *tsa1^C47S^ rad51*, *tsa1^C170S^ rad51*, or *tsa1^CCS^ rad51* double mutants grown under aerobic (upper panel) or under anaerobic conditions (bottom panel).

As a member of the 2-Cys Prx family, Tsa1 has both conserved peroxidatic Cys (Cys^47^) and resolving Cys (Cys^170^). Both Cys^47^ and Cys^170^ were shown to be necessary for the maintenance of the dimeric structure of oxidized Tsa1, and Cys^47^, but not Cys^170^, is essential for antioxidant activity measured *in vitro* [27]. To assess the effect of Cys substitutions on Tsa1 function in suppressing genome instability and cell death, we constructed isogenic mutant strains *tsa1^C47S^*, *tsa1^C170S^* and *tsa1^CCS^* that express mutant forms of Tsa1 in which Cys^47^, Cys^170^ or both were substituted with serines (Figure 2). Significantly, strains with *tsa1^C47S^* or *tsa1^CCS^* alleles had elevated mutation rates in the four mutator assays that were indistinguishable from that of the *tsa1* deletion mutant (Table 1). In contrast, the mutation rates of the *tsa1^C170S^* strain were not as elevated as seen in the *tsa1* mutant (95% confidence limits) but were higher than that of the wild-type strain. We then analyzed the meiosis products of the heterozygous diploid cells containing a *tsa1* allele of interest and a *rad51* mutation. Tetrad dissection and genotyping of viable spore colonies revealed that a *rad51* mutation was lethal in combination with the *tsa1^C47S^* or *tsa1^CCS^* alleles (Figure 4) as seen with the *tsa1* deletion (Figure 1B), while *tsa1^C170S^* allele only resulted in apparently slightly slower growth in combination with a *rad51* mutation (Figure 4). The viability and growth defects of the *tsa1^C47S^ rad51* and *tsa1^CCS^ rad51* double mutants seen under aerobic conditions were entirely restored under anaerobic conditions (Figure 4). Thus, the presence of Cys^47^ is essential for the activity of Tsa1 in mutation suppression and protection of cell death, while the role of Cys^170^ is less important in this regard.

### Cell Death Processes in the *tsa1^CCS^ rad51* Double Mutant

**Figure 5:**
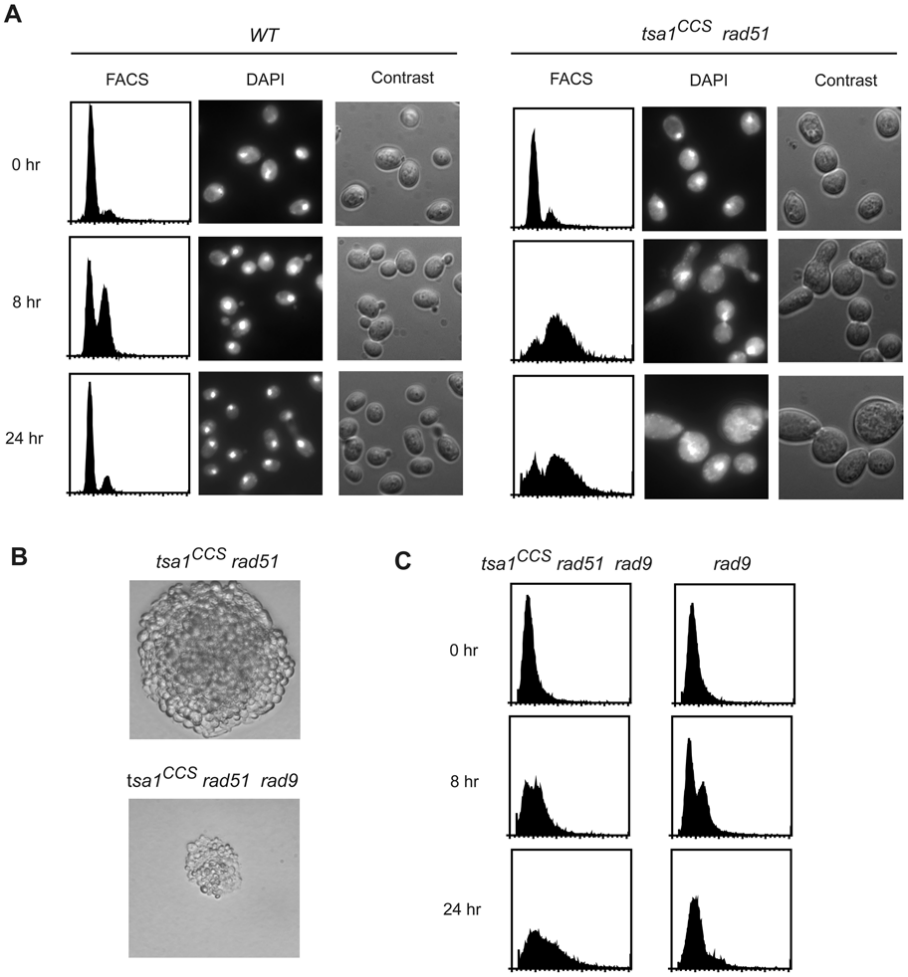
Properties of a *tsa1^CCS^ rad51* double mutant under aerobic growth. (A) Cultures of wild-type and *tsa1^CCS^ rad51* cells from anaerobic growth conditions were diluted in fresh medium and samples were taken at time 0, 8 and 24 hr after shifting to aeration to perform FACS analysis and microscopic observation by phase contrast and after DAPI staining. The same magnification (×1000) was used for microscopic analysis of both strains. The FACS profile and morphology of *rad51* and *tsa1^CCS^* cells were similar to that of wild-type (data not shown). (B) Representative microscopic images of *tsa1^CCS^ rad51* and *tsa1^CCS^ rad51 rad9* spore colonies (magnification×400) 48 hr after tetrad dissection of a *tsa1^CCS^*/*TSA1 rad51/RAD51 rad9/RAD9* heterozygous diploid (MEHY2089). The genotype of the microcolonies was inferred from the segregation patterns of the different mutations present in the diploid strain. (C) FACS analysis of *tsa1^CCS^ rad51 rad9* and *rad9* cells at 0, 8, and 24 hr after shifting to aeration.

The *tsa1^C47S^ rad51*, *tsa1^CCS^ rad51* and *tsa1 rad51* double mutants usually formed microcolonies of several hundred to several thousand cells on dissection plates, whereas colonies of wild-type or single mutant strains contained 10^7^ cells under the same conditions. This observation indicates that cells deficient in both Tsa1 peroxidase activity and recombinational repair were able to undergo a limited number of cell divisions before they stopped growing. The fact that the double mutants could grow under anaerobic condition enabled us to obtain a sufficient quantity of cells to then investigate the process of cell death under aerobic conditions in liquid culture. Colonies of *tsa1^CCS^ rad51* and control strains (wild-type, *tsa1^CCS^* and *rad51* single mutants) obtained under anaerobic conditions were inoculated into liquid cultures and grown under the same anaerobic conditions for 3 to 4 days. Cultures were diluted into fresh medium to a density of 2×10^6^ cells/ml under aerobic conditions and samples were taken for flow cytometry (FACS) and microscopic analysis at various time points after shifting to aeration. At time 0, the *tsa1^CCS^ rad51* cells exhibited cell cycle profiles and cell morphology that were similar to that of wild-type cells (Figure 5A) and to those of single mutant cells (data not shown). In contrast, 8 hr after shifting to aeration the *tsa1^CCS^ rad51* double mutants showed an abnormal cell cycle profile with a broader S/G2/M DNA content peak in the FACS profile compared to that of wild-type cells (Figure 5A). At this time point, ∼20% of wild-type cells were large-budded with a nucleus located in both the mother cell and bud whereas the *tsa1^CCS^ rad51* cells were larger and ∼80% of the cells were large-budded cells that usually had a single nucleus located at the bud neck. At 24 hr, the wild-type cells reached the beginning of stationary phase and mostly had a G1 DNA content whereas the *tsa1^CCS^ rad51* cells were predominantly large-budded cells with a single nucleus at the bud neck along with an increased number of cells with an irregular size/shape containing one or several randomly distributed nuclear fragments (Figure 5A). However, at least some of the *tsa1^CCS^ rad51* mutant cells were able to proceed past the S/G2/M arrest as the total number of cells in this culture continued to slowly increase. These observations are consistent with the idea that the *tsa1^CCS^ rad51* cells suffered rapid production of DNA damage under aerobic conditions that caused a transient or irreversible cell cycle arrest, triggering cell death. The *tsa1 rad51* double mutants displayed the same phenotypes as *tsa1^CCS^ rad51* cells (data not shown).

The *tsa1^CCS^ rad51* cells displayed a cell morphology and FACS profile suggestive of a protracted G2/M checkpoint arrest (Figure 5A). To investigate the involvement of the G2/M checkpoint, the effect of introducing a *rad9* mutation into a *tsa1 ^CCS^ rad51* strain was examined. A *tsa1^CCS^*/*TSA1 rad51*/*RAD51 rad9*/*RAD9* diploid strain was sporulated and 24 hr and 48 hr after tetrad dissection the resulting microcolonies were photographed and the genotypes of these microcolonies were inferred from mutation segregation patterns. The *tsa1^CCS^ rad51* cells formed microcolonies after 24 hr incubation and continued to grow slowly to form microcolonies composed of several hundreds to thousands of large cells (Figure 5B). The microcolonies that grew from *tsa1^CCS^ rad51 rad9* cells were usually composed of less than one hundred cells and stopped growing after 24 hr with apparent cellular degradation on further incubation (Figure 5B). In liquid culture, *tsa1^CCS^ rad51 rad9* cells obtained under anaerobic condition did not experience a delay at G2/M 8 hr after shifting to aeration (Figure 5C), in contrast with the FACS profile of *tsa1^CCS^ rad51* cells (Figure 5B). Also, the *tsa1^CCS^ rad51 rad9* cells showed a more rapid reduction of overall cell growth compared with *tsa1^CCS^ rad51* cells. These observations indicate that a Rad9-dependent checkpoint contributed to the G2/M arrest and cell survival of *tsa1^CCS^ rad51* cells.

**Figure 6:**
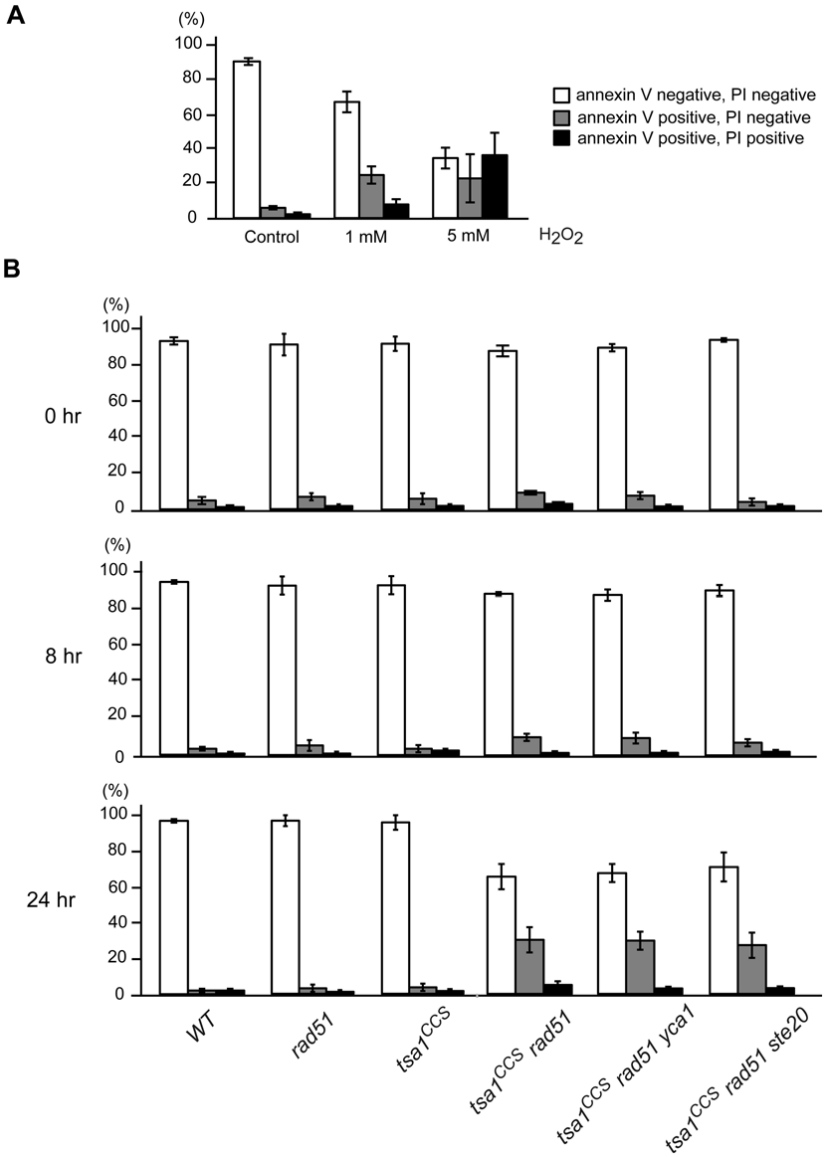
Quantification of phosphatidylserine externalization and loss of membrane integrity. (A) Exponentially growing wild-type cells were treated with 1 mM and 5 mM H_2_O_2_ for 200 min and assayed using FACS based measurement of annexin V/PI co-staining. The percentage of intact cells (double negative), early apoptotic cells (annexin V positive and PI negative) and late apoptotic/necrotic cells (double positive) is presented. (B) The indicated strains were similarly characterized at different time points after shifting from anaerobic to aerobic growth. The values presented are the mean±SD of the values from at least four independent experiments.

The *tsa1^CCS^ rad51* double mutant cells were then analyzed to determine if they suffer an apoptosis-like cell death. Phosphatidylserine externalization is considered as a typical early marker of apoptosis [28]. The simultaneous detection of phosphatidylserine externalization and loss of membrane integrity with fluorescein isothiocyanate-conjugated annexin V and propidium iodide (PI), respectively, discriminates between early apoptosis (annexin V^+^), late apoptosis eventually leading to secondary necrosis (annexin V^+^/PI^+^) and primary necrosis (PI^+^) [28],[29]. As a positive control, the isogenic wild-type strain, treated with 1 mM and 5 mM H_2_O_2_ for 200 min as previously described [30], displayed 30–50% annexin V-stained cells (Figure 6A). We then followed this apoptosis marker in wild-type and mutant cells after switching to aerobic conditions. Immediately following shifting to aeration (time 0), the wild-type and mutant cells exhibited low levels of annexin V staining (Figure 6B). The wild-type, *tsa1^CCS^* single mutant and *rad51* single mutant cells maintained low level annexin V staining at 8 hr and 24 hr after shifting to aerobic conditions (Figure 6B). In contrast, in cultures of *tsa1^CCS^ rad51* double mutants, the proportion of annexin V^+^ cells was slightly increased at the 8 hr time point after shifting to aeration and a large accumulation of annexin V^+^ cells was detected at the 24 hr time point (Figure 6B). Cultures of *tsa1 rad51* double mutant cells behaved similarly to those of *tsa1^CCS^ rad51* double mutants when they were examined under the same conditions (data not shown). Taken together, these results suggest that the *tsa1^CCS^ rad51* and *tsa1 rad51* double mutant cells undergo a DNA damage induced apoptosis-like cell death.

We next investigated whether the two independent cell death processes mediated by the proapoptotic caspase Yca1 and the Ste20 kinase, respectively, play a role in the death of *tsa1^CCS^ rad51* cells. Deletion of *YCA1* or *STE20* makes cells more resistant to H_2_O_2_ induced apoptosis [31],[32]. The diploid strains *tsa1^CCS^*/*TSA1 rad51*/*RAD51 yca1*/*YCA1* and *tsa1^CCS^*/*TSA1 rad51*/*RAD51 ste20*/*STE20* were sporulated and dissected. Tetrads were analyzed and the genotypes were determined or inferred from the mutation segregation patterns. The colony size of *tsa1^CCS^ rad51 yca1* and *tsa1^CCS^ rad51 ste20* triple mutants was similar to that of *tsa1^CCS^ rad51* double mutants (data not shown). We then tested whether the accumulation of annexin V^+^ cells in aerobic cultures of *tsa1^CCS^ rad51* mutants was Yca1- or Ste20- dependent. Anaerobic culture condition restores cellular viability of these double and triple mutants, allowing us to obtain sufficient number of cells in liquid cultures for analysis. As shown in Figure 6, both *tsa1^CCS^ rad51 yca1* and *tsa1^CCS^ rad51 ste20* cells exhibited annexin V and PI staining features that were similar to those of *tsa1^CCS^ rad51* cells at different time points after shifting to aeration. Therefore, neither deletion of *YCA1* nor deletion of *STE20* reduced the apoptotic like phenotype of *tsa1^CCS^ rad51* cells. Thus, the Yca1 and Ste20 mediated apoptosis programs do not by themselves contribute to the lethal effects caused by loss of both Tsa1 and Rad51 function. In addition, a cell-permeant pan caspase inhibitor Z-VAD-FMK failed to improve growth and survival of *tsa1^CCS^ rad51* cells (data not shown).

## Discussion

Prxs have generated considerable of interest during recent years, and understanding their distinct roles as H_2_O_2_ scavengers, redox signal transducers and molecular chaperones is of significant interest [1],[33],[34]. The distinct and overlapping subcellular localization of Prxs in *S. cerevisiae* results in a complex protein network that may reflect the particular physiological roles of each isoform. In this study, our comparative analysis of the five Prxs demonstrated that Tsa1 was distinguished from other Prxs in that by itself it played a key role in suppressing the accumulation of mutations and genome rearrangements, and maintaining cellular survival and growth in the absence of recombinational repair. The predominant role of Tsa1 in this regard may mask the potential phenotype of other *prx* mutants. Indeed, analysis of double mutants combining *tsa1* and mutation of one of the other four *PRXs* revealed distinct roles of Tsa2 and Dot5 in suppressing the accumulation of mutations and in preventing killing by exogenous oxidative stress.

In contrast to that seen for a *tsa1* mutation, a *tsa2* mutation did not cause sensitivity to H_2_O_2_, a mutator phenotype or synthetic lethality when combined with a *rad51* mutation. However, the *tsa1 tsa2* double mutant exhibited much higher H_2_O_2_ sensitivity than *tsa1* single mutant. The mutator phenotype of *tsa1 tsa2* double mutant was more pronounced than that of *tsa1* single mutant due to the increase in base substitution and frameshift mutations as revealed by different mutator assays performed in this study. Furthermore, overexpression of Tsa2 under the control of *TSA1* promoter largely restored the genetic defects caused by a *tsa1* mutation. These observations indicate that Tsa1 and Tsa2 share functional similarity in removing low endogenous concentrations of H_2_O_2_ and in dealing with more aggressive exogenous oxidant challenge. This view is consistent with the fact that Tsa2 is unique in sharing striking homology with Tsa1 among the five Prxs in *S. cerevisiae*, that Tsa1 and Tsa2 exhibit comparable antioxidant activity *in vitro* and that both have heat shock protective chaperone activity [14],[17]. While the basal transcription level of *TSA2* is much lower than that of *TSA1*, transcription of *TSA2* shows greater induction after treating cells with different oxidants, including H_2_O_2_ [3],[17]. The potently induced Tsa2 levels in response to aggressive oxidative stress could back-up Tsa1 to some degree, explaining the modest H_2_O_2_ sensitivity of the *tsa1* single mutant (Figure 1A). In contrast, the disruption of *TSA1* only results in a slight induction of *TSA2* [13], unable to compensate the defect of Tsa1 and to prevent genome instability in the absence of Tsa1. However, these two gene products may also play distinct cellular roles and be regulated by different mechanisms. Owing to its high concentration, its reactivity and numerous protein-protein interactions [34], Tsa1 could participate in many cellular reactions directly as an H_2_O_2_ scavenger and indirectly as a regulator of redox homeostasis. In this regard, it is interesting to note that mammalian PrxI and PrxII share 77% identity in amino acid sequence and are the closest homologs of Tsa1, but the biological consequences of the absence of PrxI and PrxII in higher eukaryotes are different. Notably, PrxI-deficient mice develop malignant tumors in various tissues [35], whereas PrxII-deficient mice tend to develop only red blood cell abnormalities [36]. Furthermore, expression of human PrxI in *S. cerevisiae* appears to substitute for Tsa1 whereas expression of human PrxII does not [37].

Additional evidence of cooperation among yeast Prxs is the synergistic action of Tsa1 and Dot5 in preventing GCRs. Dot5 was characterized as a functional thiol peroxidase located in the nucleus possessing mainly alkyl-hydroperoxide reductase activity *in vitro* [3] and a *dot5* mutant strain was found to have a higher sensitivity to killing by alkyl-hydroperoxide than H_2_O_2_ [21]. Therefore, the physiological significance of Dot5 was suggested to be as an antioxidant preventing DNA oxidation by alkyl-hydroperoxide that accumulates at stationary phase growth where glucose as a carbon source is depleted and *S. cerevisiae* cells begin to use fatty acids as energy sources [21]. The synergistic effect of *tsa1* and *dot5* regarding the GCR phenotype observed in the present study suggests that Tsa1 has a dominant role in protection against DNA damage leading to GCRs in the nucleus. However, Dot5 appears to be important in the absence of Tsa1. Although Tsa1 is mainly localized in the cytoplasm [3], its presence in the nucleus can not be excluded. Notably, PrxI, the mammalian functional homolog of Tsa1 [37], is localized in both the cytoplasm and nucleus [2]. Furthermore, it is unknown whether Tsa1 undergoes a change in sub-cellular localization in response to oxidative stress. Such compartment translocation is not uncommon for proteins involved in redox regulation. Either a minor presence of Tsa1 in the nucleus or its translocation from cytoplasm to nucleus in response to oxidative stress may complement *dot5* in detoxifying endogenous H_2_O_2_ and protecting the nuclear DNA from oxidative damage. In contrast to the *tsa1 tsa2* double mutant that exhibited higher H_2_O_2_ sensitivity than the *tsa1* single mutant and increased base substitution and frameshift mutations, the *tsa1 dot5* double mutant showed similar H_2_O_2_ sensitivity as the *tsa1* single mutant and increased GCRs. These notable differences may reflect the distinct and overlapping roles of each isoform in intracellular H_2_O_2_ scavenging and/or H_2_O_2_ signaling, depending on sources, intensity and targets of H_2_O_2_ in various stress conditions.

The peroxidase active site is essential for Tsa1 to function in suppressing genome instability and cell death. Tsa1^C47S^ lacking the peroxidatic cysteine and Tsa1^CCS^ lacking both the peroxidatic and resolving cysteines were non-functional as their expression resulted in the same genome instability phenotype as that caused by a *tsa1* deletion and was unable to support aerobic growth of *S. cerevisiae* cells when recombinational repair was compromised. In contrast, Tsa1^C170S^ lacking the resolving Cys retained significant function as its expression only resulted in a weak mutator phenotype and it could largely support aerobic growth of *S. cerevisiae* cells when recombinational repair was compromised. This result could be explained if Tsa1^C170S^ is able to scavenge the H_2_O_2_ generated during aerobic growth, providing evidence of significant *in vivo* peroxidase activity of a 2-Cys Prx lacking the resolving Cys. *In vitro*, the protein Tsa1^C47S^ lacks peroxidase activity regardless of whether the reducing equivalents are provided by dithiothreitol (DTT) or by the thioredoxin system, whereas Tsa1^C170S^ is active in the presence of DTT and inactive in the presence of the thioredoxin system [7],[27]. Nevertheless, Tsa1^C170S^ was only partially functional *in vivo*, as shown by the partial complementation of a *tsa1* strain expressing this protein. The basis for this activity is unclear as it is not clear what provides the needed redox system *in vivo*. Consistent with these observations, the only 2-Cys Prx of *S. pombe*, Tpx1, which is essential for cell viability, is able to support aerobic growth when the resolving Cys is eliminated in the Tpx1^C169S^ mutant protein [25]. Our results also provide *in vivo* evidence suggesting that Tsa1 may be the primary scavenger of endogenous H_2_O_2_ in *S. cerevisiae*. Several aspects of observations are consistent with this view. First, Tsa1's affinity for H_2_O_2_ with a Km of ∼12 µM [17], is similar to that of Tsa2 and Prx1 [17],[22] and is higher than that of Ahp1 [38]. But the high level of Tsa1 in the yeast cytoplasm during the full growth cycle suggests a physiological role of Tsa1 as the major scavenger [3]. Second, Tsa1 appears kinetically more efficient than other peroxide scavengers such as catalase or glutathione peroxidase in removing low endogenous concentrations of H_2_O_2_ [34]. Catalase may become more important when H_2_O_2_ concentrations are high. Third, and more important, Tsa1 is functionally unique with regard to maintenance of genome stability and to interaction with other aspects of DNA metabolism, as high rates of accumulating Can^r^ mutations and chromosomal rearrangements were found only in *tsa1* mutants and synthetic lethal interactions with *rad51* mutations were only seen with a *tsa1* mutation [18]. In this regard, mutagenesis and cell death could be viewed as an important dosimeter of endogenous oxidative stress and the activity of enzymes like Tsa1.

Individual *S. cerevisiae prx* mutants did not have growth defects and a *prx* null mutant in which *TSA1*, *TSA2*, *AHP1*, *PRX1* and *DOT5* were all deleted was still viable [19], which contrasts with the aerobic lethality of a *S. pombe* mutant lacking Tpx1 [25]. This suggests that either these mutant *S. cerevisiae* cells do not generate enough H_2_O_2_ or other reactive species to produce lethal levels of damage, another cellular antioxidant activity compensates for the loss of the Prxs, or other pathways help limit the damage and prevent cell death. Recombinational repair is such a pathway critical for cell survival and growth of *tsa1* mutants, including deletion mutants and mutants with a compromised peroxidase active site (*tsa1^C47S^* and *tsa1^CCS^*). These results confirm the view that aerobic organisms generate enough H_2_O_2_ to damage their own DNA. Scavenging enzymes and appropriate DNA repair are required for growth under aerobic conditions and Tsa1 is the dominant peroxidase activity that keeps the steady-state concentration of H_2_O_2_ at non-toxic levels. Consistent with this, *E. coli fur*, *ubiH* or *ubiE* mutations most likely lead to an increased level of ROS and cause cell death in the absence of RecA-dependent recombinational repair [39]. The *fur recA* synthetic lethality has been ascribed to elevated chromosomal fragmentation. We investigated the cell death processes of *tsa1^CCS^ rad51* and *tsa1 rad51* under aerobic conditions. Several hours after shifting from anaerobic culture to aeration, *tsa1^CCS^ rad51* cell cultures were characterized by the accumulation of cells with irregular cell size/shape, abnormal nuclear staining, and a broader S/G2/M DNA content peak. At longer time after shifting to aeration, dying cells were characterized by a significant increase in the levels of typical early markers of *S. cerevisiae* apoptosis-like cell death such as phosphatidylserine externalization. This apoptosis-like phenotype did not depend on the caspase Yca1 and Ste20 kinase, two gene products each thought to control a different apoptosis-like cell death process [31],[32]. Yca1 is the only identified caspase in *S. cerevisiae* and demonstrates enzymatic peptidase activity analogous to mammalian caspase activity [31]. However, Yca1 does not always appear to be necessary for apoptosis-like cell death in *S. cerevisiae* [40],[41]. Ste20, a homolog of human Mst1 [42], translocates into the nucleus in a caspase-independent fashion and directly phosphorylates the serine 10 of histone H2B, a process necessary for execution of apoptosis in response to H_2_O_2_ treatment [32]. Therefore, some other mechanism or machinery is likely to exist in *S. cerevisiae* that triggers the death of *tsa1^CCS^ rad51* mutants. Similarly, it is known that endogenous DNA damage, replication failure and perturbation of transcription induces an apoptosis-like death in *S. cerevisiae* [43]–[45], but the precise processes linking these early events to regulated forms of cell death are still poorly understood.

The results reported here and in previous studies can be interpreted as follows. Endogenous oxidative stress due to the absence of appropriate and sufficient Tsa1 activity induces a variety of types of DNA damage, including mutagenic, replication blocking and potential lethal DNA lesions. These lesions are repaired by diversity of mechanisms including base excision repair, nucleotide excision repair, post-replication repair, recombination and potentially other mechanisms. Unrepaired mutagenic DNA lesions cause mutations and GCRs but do not significantly affect cell survival. In contrast, in the absence of appropriate repair, a growing number of potential lethal DNA lesions persist or are converted to lethal double-strand break (DSB), activating the Rad9-dependent G2/M checkpoint. This checkpoint activation facilitates repair of at least some of the DNA damage by back-up repair mechanisms allowing checkpoint proficient cells to proliferate to a greater extent than checkpoint deficient cells. Finally, the presence of irreparable or excessive DSBs appears to initiate a DNA damage-induced apoptotic program, although how such damage signals the apoptotic program is not well understood.

## Materials and Methods

### Media and Strains

*S. cerevisiae* strains were grown in standard media including yeast extract peptone dextrose medium (YPD) or synthetic complete medium (SC) lacking appropriate amino acids as indicated. Cycloheximide resistant strains (Cyh^R^) containing mutations in the *CYH2* gene were selected on YPD agar plates containing 10 µg/ml cycloheximide. Canavanine resistant mutants (Can^r^) caused by inactivation of the *CAN1* gene were selected on SC–arginine dropout plates containing 60 mg/l canavanine. Hom3^+^ revertants were selected for on SC–threonine dropout plates and Lys2^+^ revertants were selected for on SC–lysine dropout plates. Canavanine- and 5-fluoroorotic acid (5FOA)- resistant mutants (Can^r^-5FOA^r^) resulted from the loss of the region including *CAN1* and *URA3* on chromosome V were selected on SC–arginine and –uracil dropout plates containing 60 mg/l canavanine, 1 g/l 5FOA and 50 mg/l uracil [46]. Sensitivity to H_2_O_2_ was assayed on solid media by spotting 10-fold serial dilutions of mid-logarithmic phase cells onto SC plates containing H_2_O_2_ at indicated concentrations.

The strains used for this study were all isogenic to the S288c based parental strain RDKY3615 *MATa*, *ura3-52*, *leu2Δ1*, *trp1Δ63*, *his3Δ200*, *lys2ΔBgl*, *hom3-10*, *ade2Δ1*, *ade8*, *hxt13*::*URA3* [47] (Table 2). Gene replacements were made using standard PCR-based homology-directed methods [48]. Double or triple mutation combinations were usually constructed by crossing of two haploids or modifying a target gene in a diploid strain followed by tetrad dissection. The construction of the *tsa1*::*TSA2* mutant in which the entire *TSA1* gene was replaced by *TSA2* coding sequence under control of the native *TSA1* promoter is illustrated schematically in Figure 2. The recipient strain MEHY1539 (Trp^+^ Cyh^S^) in which the entire *TSA1* gene was replaced by the *TRP1-CYH2* cassette as previously described [37]. PCR-generated fragments containing *TSA2* flanked by sequences homologous to the upstream and downstream sequence of *TSA1*, were used to transform MEHY1539 (Trp^+^ Cyh^S^) to replace *TRP1-CYH2* at the *TSA1* locus yielding the strain MEHY1710 (Trp^−^ Cyh^R^) in which the *TSA2* is under the control of the native *TSA1* promoter. Correct integration of *TSA2* at *TSA1* locus was confirmed by genomic PCR and DNA sequencing.

**Table 2:** *S. cerevisiae* strains used in this study.

Strain	Genotype or construction	Source
RDKY3615	*MAT* **a** *ura3-52 leu2Δ1 trp1Δ63 his3Δ200 lys2ΔBgl hom3-10*, *ade2Δ1*, *ade8*, *hxt13Δ*::*URA3*	[47]
RDKY5502	RDKY3615 with *tsa1*::*kanMX4*	[20]
RDKY5653	RDKY3615 with *tsa2*::*TRP1*	This study
RDKY5654	RDKY3615 with *ahp1*::*TRP1*	This study
RDKY5655	RDKY3615 with *prx1*::*TRP1*	This study
RDKY5656	RDKY3615 with *dot5*::*TRP1*	This study
RDKR3636	RDKY3615 with *rad51*::*HIS3*	[20]
RDKY5696	RDKY3615 with *tsa1*::*kanMX4 tsa2*::*TRP1*	This study
RDKY5697	RDKY3615 with *tsa1*::*kanMX4 ahp1*::*TRP1*	This study
RDKY5698	RDKY3615 with *tsa1*::*kanMX4 prx1*::*TRP1*	This study
MEHY793	RDKY3615 with *tsa1*::*kanMX4 dot5*::*TRP1*	This study
MEHY694	*MAT* **α** *rad51*::*HIS3*	This study
MEHY575	*MAT* **α** *rad6*::*HIS3*	This study
MEHY101	RDKY5502×MEHY694	This study
MEHY591	RDKY5502×MEHY575	This study
MEHY1853	RDKY5653×MEHY694	This study
MEHY1922	RDKY5654×MEHY694	This study
MEHY1923	RDKY5655×MEHY694	This study
MEHY1925	RDKY5656×MEHY694	This study
MEHY1537	RDKY3615 with *cyh2*	[37]
MEHY1539	MEHY1537 with *tsa1*::*TRP1-CYH2*	[37]
MEHY1710	MEHY1537 with *tsa1*::*TSA2*	This study
MEHY1838	MEHY1710×MEHY694	This study
MEHY1841	MEHY1710×MEHY575	This study
MEHY1746	RDKY3615 with *TSA1-3HA TRP1*	[37]
MEHY2174	RDKY3615 with *TSA2-3HA TRP1*	This study
MEHY2274	MEHY1710 with *tsa1*::*TSA2-3HA TRP1*	This study
MEHY1702	MEHY1537 with *tsa1^C47S^*	This study
MEHY1593	MEHY1537 with *tsa1^C170S^*	This study
MEHY1590	MEHY1537 with *tsa1^CCS^*	This study
MEHY1844	MEHY1702×MEHY694	This study
MEHY1847	MEHY1593×MEHY694	This study
MEHY1850	MEHY1590×MEHY694	This study
MEHY1891	MEHY1537 with *tsa1^CCS^ rad51*::*HIS3*	This study
MEHY2089	MEHY1850 with *rad9*::*TRP1*	This study
MEHY2131	MEHY1537 with *tsa1^CCS^ rad51*::*HIS3 rad9*::*TRP1*	This study
MEHY1988	MEHY1850 with *yca1*::*TRP1*	This study
MEHY2069	MEHY1537 with *tsa1^CCS^ rad51*::*HIS3 yca1*::*TRP1*	This study
MEHY1981	MEHY1850 with *ste20*::*TRP1*	This study
MEHY2067	MEHY1537 with *tsa1^CCS^ rad51*::*HIS3 ste20*::*TRP1*	This study

The strains used for this study are all isogenic to the S288c based parental strain RDKY3615. All mutants are deletion mutation except for the mutant strains *tsa1^C47S^*, *tsa1^C170S^*, and *tsa1^CCS^* that express point-mutated Tsa1 proteins.

Strains with mutated versions of *TSA1* (*tsa1^C47S^*, *tsa1^C170S^*, and *tsa1^CCS^* which contain the C47S, C170S, or both C47S and C170S substitutions, respectively) were created as follows. The *TSA1* gene with 5′ and 3′ flanking DNA sequence was amplified from genomic DNA using primers containing, respectively, the restriction sites *Xho*I and *Hind*III. The resulting PCR product was digested with *Xho*I and *Hind*III and cloned in *Xho*I and *Hind*III sites of the plasmid pRS416 [49]. Site-directed mutagenesis to produce the C47S, C170S and both C47S and C170S substitutions was done with the QuickChange II Site-Directed Mutagenesis Kit (Strategene) and mutagenic primers 5′-GCCTTCACTTTCGTCTCTCCAACCGAAATCATTGC-3′ and 5′-GCAATGATTTCGGTTGGAGAGACGAAAGTGAAGGC-3′ (sequences for Cys^47^ to Ser substitution are underlined), and mutagenic primers 5′-GGTACTGTCTTGCCATCTAACTGGACTCCAGGTGC-3′ and 5′-GCACCTGGAGTCCAGTTAGATGGCAAGACAGTACC-3′ (sequences for Cys^170^ to Ser substitution are underlined). The mutated *TSA1* gene was sequenced to verify the presence of the desired substitutions and the absence of other mutations. PCR-generated fragments containing mutant alleles of *TSA1* flanked by sequences homologous to the upstream and downstream sequence of *TSA1*, were used to transform MEHY1539 (Trp^+^ Cyh^S^) to replace *TRP1-CYH2* at the *TSA1* locus yielding the strains MEHY1702 (*tsa1^C47S^*), MEHY1593 (*tsa1^C170S^*) and MEHY1590 (*tsa1^CCS^*), expressing a given mutant allele of *TSA1* under the control of the native *TSA1* promoter (Figure 2). Correct integration of mutant alleles of *TSA1* at the wild-type *TSA1* locus was confirmed by genomic PCR and DNA sequencing.

The *S. cerevisiae* strains expressing HA epitope-tagged Tsa1 and Tsa2 at their native loci or Tsa2 produced under the control of the *TSA1* promoter were constructed as previously described [37]. Each forward primer has a shared 3′ end that allows for PCR amplification of a 3*HA*-*TRP1* cassette in the plasmid pFA6a-3*HA*-*TRP1* [50], as well as a gene-specific 5′ end that allows for precise integration of the amplified cassette at the 3′ end of the genomic coding sequence through homologous recombination. The reverse primer has 3′ end complementary to the 3*HA*-*TRP1* cassette for PCR amplification and a 5′ end homologous to the downstream sequence of the coding region of each gene. These amplified cassettes were used to transform strains RDKY3615 or MEHY1710 followed by selection on SC–tryptophan dropout plates. Insertion of the cassette and in-frame fusion of the 3HA tag at the C-terminal end of the coding region of each gene was confirmed by genomic PCR and sequencing. Sequences of primers used in strain constructions are available on request.

### Quantitative RT–PCR and Western Blot

Wild-type and *tsa1*::*TSA2* strains were grown to mid-exponential phase in YPD medium. Total RNA was extracted with acidic phenol (pH 5.0) according to an established procedure [51]. Extracted RNA was treated with DNase and verified by conventional PCR to ensure the absence of trace DNA in the samples. cDNA was generated using an iScript cDNA synthesis Kit (Bio-Rad). We designed gene-specific primers for *TSA1*, *TSA2*, and *ADH1* (encoding alcohol dehydrogenase and used as an internal control). Serial dilutions of cDNA from each strain were amplified using the appropriate primers and iQ SYBR Green Supermix (Bio-Rad). A single melt curve peak was observed for each sample used in data analysis, thus confirming the purity and specificity of all amplified products. The relative ratio of *TSA1* or *TSA2* expression to *ADH1* was calculated using the relative quantity (ΔΔCT) analysis formula in the Bio-Rad iQ5 Gene Expression Optical System Software.

For Western blots, strains were grown to mid-logarithmic phase in YPD medium and whole cell extracts isolated by trichloroacetic acid extraction [52]. Protein concentrations were measured by the Bradford procedure (Bio-Rad) using bovine serum albumin as a standard. Cell extracts were electrophoresed under reducing conditions on 12% SDS-PAGE minigels and electrophoretically transferred to nitrocellulose membrane that was probed with the mouse anti-HA (Roche Diagnostics) or rabbit anti-yeast alcohol dehydrogenase (anti-Adh, Chemicon International). Corresponding horseradish peroxidase-conjugated secondary antibody was detected with the Amersham ECL Western blotting analysis system (GE Healthcare).

### Measurement of Mutation Rates

The rate of accumulation of Can^r^ mutations, Hom3^+^ revertants, Lys2^+^ revertants, and GCRs in cell populations was determined by fluctuation analysis as described previously [20]. For each strain, cells were diluted to approximately 100 cells/ml in at least 15 independent YPD cultures of 2 to 50 ml, depending on the mutator assay and strain, grown to 1–2×10^8^ cells/ml, harvested, washed and resuspended in sterile water. Sufficient quantities of cells were plated on appropriate selection media to identify Can^r^ mutations, Hom3^+^ revertants, Lys2^+^ revertants and GCR events, and appropriate dilutions were plated on YPD for total cell counts. Colonies were counted after four days of growth at 30°C. The number of mutant cells per culture was calculated and the median value from at least 15 independent cultures for each strain was used to determine the mutation rate as described [53]. The 95% confidence intervals for a median rate were calculated based on order statistics with the formula available at http://www.math.unb.ca/~knight/utility/MedInt95.htm.

### Anaerobic Growth Conditions

YPD medium with or without agar was supplemented with Tween 80 and ergosterol to a final concentration of 1.32 ml/l and 6.75 mg/l, respectively. Anaerobic liquid cultures or anaerobic growth of dissected spores in plates were placed in an airtight jar containing a disposable hydrogen- and carbon dioxide-generating envelope (BBL GasPak Plus) and grown anaerobically at 30°C for 3–5 days to yield enough cells for analysis. Anaerobic conditions were monitored with the redox indicator Anaerotest (Merck) placed inside of the airtight jar.

### DNA Content Analysis and 4,6-diamidino-2-phenylindole (DAPI) Staining

Cells of the desired genotype were grown in liquid culture under same anaerobic condition for 3 to 4 days. Cultures were diluted in fresh medium to a density of 2×10^6^ cells/ml and samples were taken at various time points after shifting to aeration. Cells were collected, resuspended in 70% (vol/vol) ethanol for fixation. The cells were washed in 50 mM Tris buffer (pH 8.0), incubated 24 hr with 5 mg/ml DNase-free RNase, centrifuged and labeled with 50 µg/ml PI, sonicated and submitted to FACS measurements of DNA content with a FACScalibur cytometer (Beckton-Dickinson). Same fixed cells were also stained with DAPI [48]. Fluorescent and phase contrast images were captured with a Leica microscope (DMRXA) equipped with a cooled CCD camera MicroMAX (Princeton Instruments) under control of the MetaMorph software (Molecular Devices). Images obtained were processed with software ImageJ.

### Annexin V and PI Staining

Annexin V/PI co-labeling was performed using the Vybrant Apoptosis Assay kit #3 (Invitrogen) according to the manufacturer's instructions, excepted that about 1×10^6^ intact yeast living cells were treated by 5% glusulase (Perkin Elmer) and 5 U/ml lyticase (Bio-Rad) at 28°C for 2 hr in a buffer consisting of 50 mM HEPES, 700 mM NaCl, 12.5 mM CaCl_2_, pH 7.4. The labeled cells were then analyzed by FACS using an argon-ion laser (excitation wavelength 488 nm and emission of 530 nm) and the data were processed using BD Cell Quest Pro software (version 5.2).

## References

[1] Rhee SG, Chae HZ, Kim K (2005). Peroxiredoxins: a historical overview and speculative preview of novel mechanisms and emerging concepts in cell signaling.. Free Radic Biol Med.

[2] Wood ZA, Schroder E, Robin Harris J, Poole LB (2003). Structure, mechanism and regulation of peroxiredoxins.. Trends Biochem Sci.

[3] Park SG, Cha MK, Jeong W, Kim IH (2000). Distinct physiological functions of thiol peroxidase isoenzymes in *Saccharomyces cerevisiae*.. J Biol Chem.

[4] Poole LB (2005). Bacterial defenses against oxidants: mechanistic features of cysteine-based peroxidases and their flavoprotein reductases.. Arch Biochem Biophys.

[5] Biteau B, Labarre J, Toledano MB (2003). ATP-dependent reduction of cysteine-sulphinic acid by *S. cerevisiae* sulphiredoxin.. Nature.

[6] Chang TS, Jeong W, Woo HA, Lee SM, Park S (2004). Characterization of mammalian sulfiredoxin and its reactivation of hyperoxidized peroxiredoxin through reduction of cysteine sulfinic acid in the active site to cysteine.. J Biol Chem.

[7] Chae HZ, Chung SJ, Rhee SG (1994). Thioredoxin-dependent peroxide reductase from yeast.. J Biol Chem.

[8] Lee J, Spector D, Godon C, Labarre J, Toledano MB (1999). A new antioxidant with alkyl hydroperoxide defense properties in yeast.. J Biol Chem.

[9] Inoue Y, Matsuda T, Sugiyama K, Izawa S, Kimura A (1999). Genetic analysis of glutathione peroxidase in oxidative stress response of *Saccharomyces cerevisiae*.. J Biol Chem.

[10] Avery AM, Avery SV (2001). *Saccharomyces cerevisiae* expresses three phospholipid hydroperoxide glutathione peroxidases.. J Biol Chem.

[11] Tanaka T, Izawa S, Inoue Y (2005). GPX2, encoding a phospholipid hydroperoxide glutathione peroxidase homologue, codes for an atypical 2-Cys peroxiredoxin in *Saccharomyces cerevisiae*.. J Biol Chem.

[12] Maiorino M, Ursini F, Bosello V, Toppo S, Tosatto SC (2007). The thioredoxin specificity of *Drosophila* GPx: a paradigm for a peroxiredoxin-like mechanism of many glutathione peroxidases.. J Mol Biol.

[13] Wong CM, Zhou Y, Ng RW, Kung Hf HF, Jin DY (2002). Cooperation of yeast peroxiredoxins Tsa1p and Tsa2p in the cellular defense against oxidative and nitrosative stress.. J Biol Chem.

[14] Jang HH, Lee KO, Chi YH, Jung BG, Park SK (2004). Two enzymes in one; two yeast peroxiredoxins display oxidative stress-dependent switching from a peroxidase to a molecular chaperone function.. Cell.

[15] Monje-Casas F, Michan C, Pueyo C (2004). Absolute transcript levels of thioredoxin- and glutathione-dependent redox systems in *Saccharomyces cerevisiae*: response to stress and modulation with growth.. Biochem J.

[16] Ghaemmaghami S, Huh WK, Bower K, Howson RW, Belle A (2003). Global analysis of protein expression in yeast.. Nature.

[17] Munhoz DC, Netto LE (2004). Cytosolic thioredoxin peroxidase I and II are important defenses of yeast against organic hydroperoxide insult: catalases and peroxiredoxins cooperate in the decomposition of H_2_O_2_ by yeast.. J Biol Chem.

[18] Huang ME, Rio AG, Nicolas A, Kolodner RD (2003). A genomewide screen in *Saccharomyces cerevisiae* for genes that suppress the accumulation of mutations.. Proc Natl Acad Sci U S A.

[19] Wong CM, Siu KL, Jin DY (2004). Peroxiredoxin-null yeast cells are hypersensitive to oxidative stress and are genomically unstable.. J Biol Chem.

[20] Huang ME, Kolodner RD (2005). A biological network in *Saccharomyces cerevisiae* prevents the deleterious effects of endogenous oxidative DNA damage.. Mol Cell.

[21] Cha MK, Choi YS, Hong SK, Kim WC, No KT (2003). Nuclear thiol peroxidase as a functional alkyl-hydroperoxide reductase necessary for stationary phase growth of *Saccharomyces cerevisiae*.. J Biol Chem.

[22] Pedrajas JR, Miranda-Vizuete A, Javanmardy N, Gustafsson JA, Spyrou G (2000). Mitochondria of *Saccharomyces cerevisiae* contain one-conserved cysteine type peroxiredoxin with thioredoxin peroxidase activity.. J Biol Chem.

[23] Veal EA, Findlay VJ, Day AM, Bozonet SM, Evans JM (2004). A 2-Cys peroxiredoxin regulates peroxide-induced oxidation and activation of a stress-activated MAP kinase.. Mol Cell.

[24] Vivancos AP, Castillo EA, Biteau B, Nicot C, Ayte J (2005). A cysteine-sulfinic acid in peroxiredoxin regulates H_2_O_2_-sensing by the antioxidant Pap1 pathway.. Proc Natl Acad Sci U S A.

[25] Jara M, Vivancos AP, Calvo IA, Moldon A, Sanso M (2007). The peroxiredoxin Tpx1 is essential as a H_2_O_2_ scavenger during aerobic growth in fission yeast.. Mol Biol Cell.

[26] Ragu S, Faye G, Iraqui I, Masurel-Heneman A, Kolodner RD (2007). Oxygen metabolism and reactive oxygen species cause chromosomal rearrangements and cell death.. Proc Natl Acad Sci U S A.

[27] Chae HZ, Uhm TB, Rhee SG (1994). Dimerization of thiol-specific antioxidant and the essential role of cysteine 47.. Proc Natl Acad Sci U S A.

[28] Bossy-Wetzel E, Green DR (2000). Detection of apoptosis by annexin V labeling.. Methods Enzymol.

[29] Frohlich KU, Fussi H, Ruckenstuhl C (2007). Yeast apoptosis—from genes to pathways.. Semin Cancer Biol.

[30] Madeo F, Frohlich E, Ligr M, Grey M, Sigrist SJ (1999). Oxygen stress: a regulator of apoptosis in yeast.. J Cell Biol.

[31] Madeo F, Herker E, Maldener C, Wissing S, Lachelt S (2002). A caspase-related protease regulates apoptosis in yeast.. Mol Cell.

[32] Ahn SH, Cheung WL, Hsu JY, Diaz RL, Smith MM (2005). Sterile 20 kinase phosphorylates histone H2B at serine 10 during hydrogen peroxide-induced apoptosis in *S. cerevisiae*.. Cell.

[33] D'Autreaux B, Toledano MB (2007). ROS as signalling molecules: mechanisms that generate specificity in ROS homeostasis.. Nat Rev Mol Cell Biol.

[34] Fourquet S, Huang ME, D'Autreaux B, Toledano MB (2008). The dual functions of thiol-based peroxidases in H_2_O_2_ scavenging and signaling.. Antioxid Redox Signal.

[35] Neumann CA, Krause DS, Carman CV, Das S, Dubey DP (2003). Essential role for the peroxiredoxin Prdx1 in erythrocyte antioxidant defence and tumour suppression.. Nature.

[36] Lee TH, Kim SU, Yu SL, Kim SH, Park DS (2003). Peroxiredoxin II is essential for sustaining life span of erythrocytes in mice.. Blood.

[37] Iraqui I, Faye G, Ragu S, Masurel-Heneman A, Kolodner RD (2008). Human peroxiredoxin PrxI is an orthologue of yeast Tsa1, capable of suppressing genome instability in *Saccharomyces cerevisiae*.. Cancer Res.

[38] Jeong JS, Kwon SJ, Kang SW, Rhee SG, Kim K (1999). Purification and characterization of a second type thioredoxin peroxidase (type II TPx) from *Saccharomyces cerevisiae*.. Biochemistry.

[39] Kouzminova EA, Rotman E, Macomber L, Zhang J, Kuzminov A (2004). RecA-dependent mutants in *Escherichia coli* reveal strategies to avoid chromosomal fragmentation.. Proc Natl Acad Sci U S A.

[40] Buttner S, Bitto A, Ring J, Augsten M, Zabrocki P (2008). Functional mitochondria are required for alpha-synuclein toxicity in aging yeast.. J Biol Chem.

[41] Guscetti F, Nath N, Denko N (2005). Functional characterization of human proapoptotic molecules in yeast *S. cerevisiae*.. FASEB J.

[42] Cheung WL, Ajiro K, Samejima K, Kloc M, Cheung P (2003). Apoptotic phosphorylation of histone H2B is mediated by mammalian sterile twenty kinase.. Cell.

[43] Weinberger M, Ramachandran L, Feng L, Sharma K, Sun X (2005). Apoptosis in budding yeast caused by defects in initiation of DNA replication.. J Cell Sci.

[44] Qi H, Li TK, Kuo D, Nur EKA, Liu LF (2003). Inactivation of Cdc13p triggers MEC1-dependent apoptotic signals in yeast.. J Biol Chem.

[45] Mazzoni C, Mancini P, Verdone L, Madeo F, Serafini A (2003). A truncated form of KlLsm4p and the absence of factors involved in mRNA decapping trigger apoptosis in yeast.. Mol Biol Cell.

[46] Schmidt KH, Pennaneach V, Putnam CD, Kolodner RD (2006). Analysis of gross-chromosomal rearrangements in *Saccharomyces cerevisiae*.. Methods Enzymol.

[47] Chen C, Kolodner RD (1999). Gross chromosomal rearrangements in *Saccharomyces cerevisiae* replication and recombination defective mutants.. Nat Genet.

[48] Amberg DC, Burke DJ, Strathern JN (2005). Methods in yeast genetics.

[49] Sikorski RS, Hieter P (1989). A system of shuttle vectors and yeast host strains designed for efficient manipulation of DNA in *Saccharomyces cerevisiae*.. Genetics.

[50] Longtine MS, McKenzie A, Demarini DJ, Shah NG, Wach A (1998). Additional modules for versatile and economical PCR-based gene deletion and modification in *Saccharomyces cerevisiae*.. Yeast.

[51] Ausubel FM, Brent R, Kingston RE, Moore DD, Seidman JG (2006). Current protocols in molecular biology.

[52] Foiani M, Marini F, Gamba D, Lucchini G, Plevani P (1994). The B subunit of the DNA polymerase alpha-primase complex in *Saccharomyces cerevisiae* executes an essential function at the initial stage of DNA replication.. Mol Cell Biol.

[53] Lea DE, Coulson CA (1948). The distribution of the numbers of mutants in bacterial populations.. J Genet.

